# Recurrent genetic defects on chromosome 5q in myeloid neoplasms

**DOI:** 10.18632/oncotarget.14130

**Published:** 2016-12-23

**Authors:** Naoko Hosono, Hideki Makishima, Reda Mahfouz, Bartlomiej Przychodzen, Kenichi Yoshida, Andres Jerez, Thomas LaFramboise, Chantana Polprasert, Michael J Clemente, Yuichi Shiraishi, Kenichi Chiba, Hiroko Tanaka, Satoru Miyano, Masashi Sanada, Edward Cui, Amit K Verma, Michael A McDevitt, Alan F List, Yogen Saunthararajah, Mikkael A Sekeres, Jacqueline Boultwood, Seishi Ogawa, Jaroslaw P Maciejewski

**Affiliations:** ^1^ Department of Translational Hematology and Oncology Research, Taussig Cancer Institute, Cleveland Clinic, Cleveland, OH, USA; ^2^ First Department of Internal Medicine, Faculty of Medical Sciences, University of Fukui, Fukui, Japan; ^3^ Cancer Genomics Project, Graduate School of Medicine, The University of Tokyo, Tokyo, Japan; ^4^ Department of Pathology and Tumor Biology, Graduate School of Medicine, Kyoto University, Kyoto, Japan; ^5^ Department of Genetics and Genome Sciences, Case Western Reserve University, Cleveland, OH, USA; ^6^ Laboratory of DNA Information Analysis, Human Genome Center, Institute of Medical Science, The University of Tokyo, Tokyo, Japan; ^7^ Laboratory of Sequence Analysis, Human Genome Center, Institute of Medical Science, The University of Tokyo, Tokyo, Japan; ^8^ Department of Oncology, Albert Einstein College of Medicine, Bronx, NY, USA; ^9^ Division of Hematology and Hematological Malignancy, Department of Internal Medicine and Oncology, Johns Hopkins University School of Medicine, Baltimore, MD, USA; ^10^ H. Lee Moffitt Cancer Center and Research Institute, Tampa, FL, USA; ^11^ Leukemia Program, Cleveland Clinic, Taussig Cancer Institute, Cleveland, OH, USA; ^12^ Department of Hematology and Medical Oncology, Taussig Cancer Institute, Cleveland Clinic, Cleveland, OH, USA; ^13^ LLR Molecular Haematology Unit, Nuffield Division of Clinical Laboratory Sciences, Radcliffe Department of Medicine, University of Oxford, John Radcliffe Hospital, Oxford, United Kingdom

**Keywords:** MDS, del(5q), TP53, G3BP1

## Abstract

**Background:**

Deletion of chromosome 5q (del(5q)) is the most common karyotypic abnormality in myeloid neoplasms.

**Materials and Methods:**

To define the pathogenic molecular features associated with del(5q), next–generation sequencing was applied to 133 patients with myeloid neoplasms (MDS; *N* = 69, MDS/MPN; *N* = 5, sAML; *N* = 29, pAML; *N* = 30) with del(5q) as a sole abnormally or a part of complex karyotype and results were compared to molecular features of patients diploid for chr5.

**Findings:**

A number of 5q genes with haploinsufficient expression and/or recurrent somatic mutations were identified; for these genes, *CSNK1A1* and *G3BP1* within the commonly deleted 5q region and *DDX41* within a commonly retained region were most commonly affected by somatic mutations. These genes showed consistent haploinsufficiency in deleted cases; low expression/mutations of *G3BP1* or *DDX41* were associated with poor survival, likely due to decreased cellular function. The most common mutations on other chromosomes in patients with del(5q) included *TP53*, and mutations of *FLT3* (ITD or TKD), *NPM1* or *TET2* and were mutually exclusive. Serial sequencing allowed for definition of clonal architecture and dynamics, in patients with exome sequencing allelic imbalance for informative SNPs facilitated simultaneous approximation of clonal size of del(5q) and clonal burden for somatic mutations.

**Interpretation:**

Our results illuminate the spectrum of molecular defects characteristic of del(5q), their clinical impact and succession of stepwise evolution.

## INTRODUCTION

Interstitial deletion of the long arm of chromosome 5 (del(5q)), is the most common karyotypic abnormality in myeloid neoplasms, observed in 10–15% of patients with myelodysplastic syndromes (MDS) [1–5] or primary acute myeloid leukemia (pAML) [6, 7], and in up to 40% of secondary myeloid leukemias (sAML) [8]. While a smaller fraction of more homogenous patients with the isolated del(5q) and classical 5q- syndrome show more favorable prognosis [9, 10], the majority of myeloid neoplasms with del(5q) are morphologically heterogeneous, and have additional cytogenetic abnormalities [7, 11, 12].

The commonly deleted regions (CDR) in del(5q) have been extensively studied with a distal region (CDR1:5q32-33) often deleted in the 5q- syndrome [2] and a proximal region (CDR2:5q31) in higher-risk MDS and AML [13]. SNP-array-based karyotyping helped to further refine the boundaries of the CDR (CDR1;145,299,747-153,828,955 and CDR2;137,500,665-139,471,723), and commonly retained regions (CRR1;from the centromere to 5q14.2 and CRR2;from 5q34 to the telomere) [5]. Patients with small interstitial deletions were shown to have a better outcome as compared to those with larger deletions [5]. Haploinsufficiency of several genes in the CDRs likely contributes to specific phenotypic features in del(5q). For instance, heterozygous deletions resulting in haploinsufficient expression of *RPS14* is a key determinant of ineffective erythropoiesis [14], while thrombocytosis and megakaryocytic dysplasia may be related to haploinsufficient *miR-145*/*miR-146a* [15]. However, experimental knockdown of these genes did not result in a growth advantage and haploinsufficiency is not uniformly present in all cases to explain clonal dominance. Recurrent hemizygous mutations of genes within the deleted locus have not been identified with the notable exception *CSNK1A1* missense mutations found in only 3/40 del(5q) cases, but absent in heterozygous configuration [16]. Moreover, most of del(5q) cases involve large deletions encompassing both CDRs, and thus identification of genes contributing to individual clinical phenotypes has been challenging. It is likely that pathogenetic mechanisms in del(5q) may involve hemizygous mutations or haploinsufficiency and be modified by additional somatic lesions affecting genes on other chromosomes. Furthermore, the position of del(5q) within the clonal hierarchy might also affect the phenotype and clinical behavior.

To characterize the genetic and genomic complexity and clonal hierarchy in myeloid neoplasms with 5q abnormalities, we used next generation sequencing (NGS), including whole exome sequencing (WES) and targeted multiamplicon deep sequencing in a cohort of patients with del(5q) in a comparison to patients with diploid chr5. In addition, to explore ancestral events in del(5q), we compared clonal size of individual somatic mutations with that of del(5q) identified by WES.

## RESULTS

### Detection of mutations within CDRs and CRRs in del(5q)

**Figure 1:**
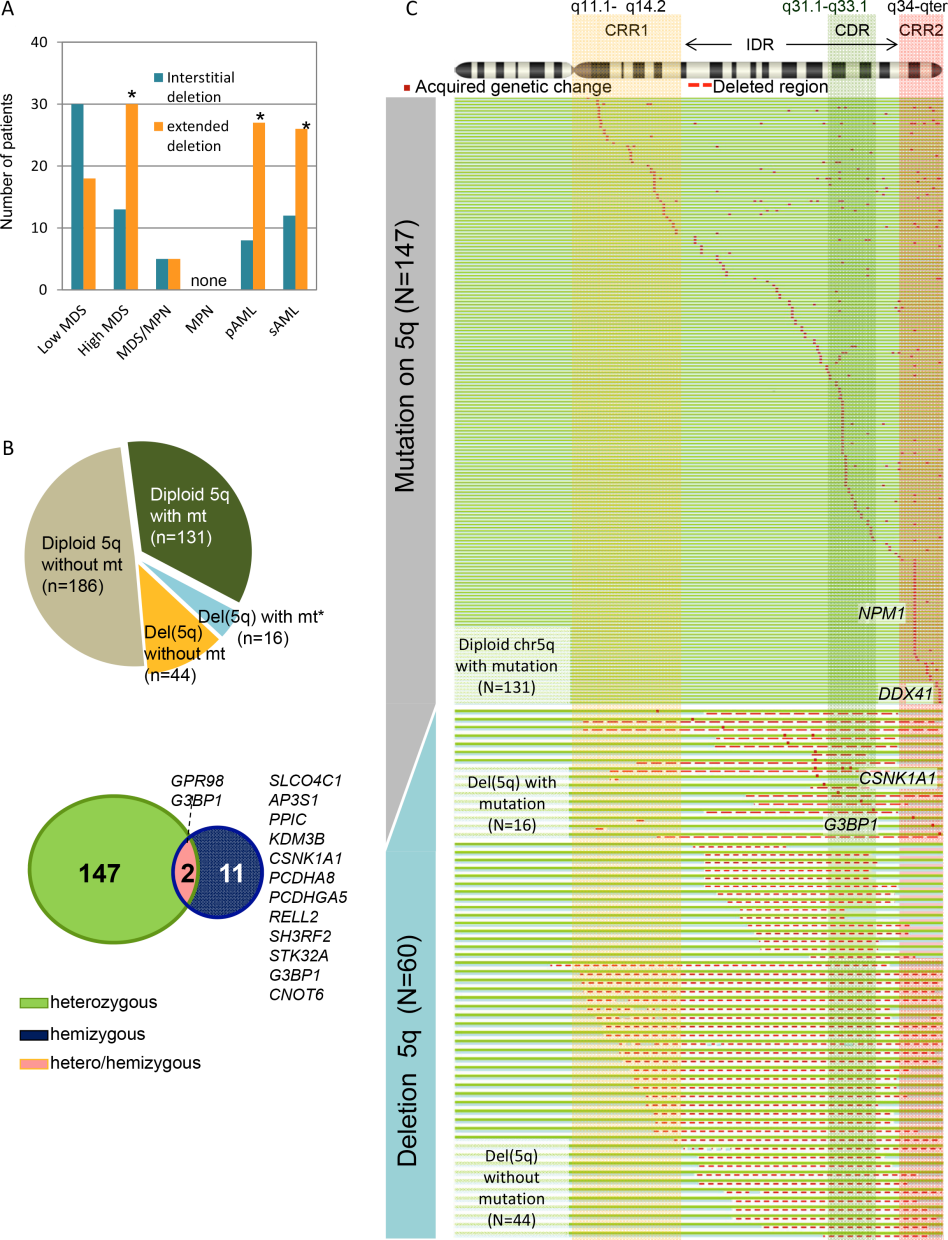
Whole spectrum of deletion 5q (del(5q)) and somatic mutations on chromosome 5q (**A**) Number of various types of del(5q) lesions affecting interstitial and commonly retained regions (CRRs) in each disease phenotype. For detection of del(5q), SNP-array karyotyping and metaphase cytogenetics were applied (*N* = 1476). interstitial deletion: deletions within 5q14.2 to 5q34, extended deletion: deletions involving the CRR1 and/or CRR2, Low-risk MDS includes: refractory cytopenia with unilineage dysplasia, refractory cytopenia with multilineage dysplasia, MDS with isolated del(5q), MDS unclassifiable, and refractory anemia with ring sideroblasts. High-risk MDS includes refractory anemia with excess blasts. *P < .001. (**B**) Frequency of cases with mutations (mt) on chromosome 5 (chr5) in each copy-number status (diploid or deletion) as demonstrated by pie chart. *In a case with del(5q), a heterozygous mutation of a gene located on the non-deleted region of chr5 was identified. Venn diagram showing the number of mutated genes categorized by their zygosity (heterozygous, hemizygous, hemizygous and heterozygous). (**C**) Mutations on chr5 detected by whole-exome sequencing (*N* = 389) were shown in red dots (see Supplementary Table S3 for individual genes). Copy number status of chr5 was demonstrated as follows: diploid 5q, green and blue lines; and del(5q), a red dashed line. Two commonly retained regions (CRR1 and CRR2) and a commonly deleted region (CDR), defined by SNP-A karyotyping analyses, are represented by vertical rectangles.

Using a combination of cytogenetic methods, including SNP-array karyotyping, FISH analysis and/or metaphase cytogenetics, we analyzed 1472 patients with myeloid neoplasms and identified various types of del(5q) in 178 (12%, isolated del(5q) *N* = 43, del(5q) with additional chromosome abnormality *N* = 135) patients and 5q uniparental disomy (UPD) in 8 (0.5%) (Table 1). After we defined large proximal (CRR1 5q11.1 to 5q14.2, 48400001-81634579) and distal (CRR2 5q34 to 5q35.3, 164213764-180915260) commonly retained genomic segments, as well as a region including the well-known CDR (5q31.1-5q33.1) as an interstitial deleted region (IDR) (5q14.2 to 5q34, 81634580-164213763), we correlated clinical characteristics with the extent of the deleted area. Interstitial deletions were frequently seen in low-risk MDS, while deletions involving the CRR1 and/or CRR2 (extended deletions) were more prevalent in high-risk MDS (RAEB1,RAEB2) and in AML compared to low-risk MDS (RCUD, RCMD, 5q-, MDS-U, RARS) (*P* < .0001, Figure 1A). No del(5q) case was observed within the MPN cohort (*N* = 81).

**Table 1:** Analysis cohort and frequency of 5q deletions and loss of heterozygosity

MDS-Low	Total	316	
(RCUD / RCMD / 5q- / MDS-U / RARS)	del(5q)		49
	UPD5q		3
	total 5q abnormality	**51**	(16%)
**MDS-High**	Total	**192**	
(RAEB 1/2)	del(5q)		44
	UPD5q		2
	total 5q abnormality	**46**	(24%)
**MDS/MPN**	Total	**259**	
	del(5q)		10
	UPD5q		1
	total 5q abnormality	**11**	(4%)
**MPN**	Total	**81**	
	del(5q)		0
	UPD5q		0
	total 5q abnormality	**0**	(0%)
**sAML**	Total	**233**	
	del(5q)		40
	UPD5q		1
	total 5q abnormality	**41**	(18%)
**pAML**	Total	**391**	
	del(5q)		35
	UPD5q		1
	total 5q abnormality	**36**	(9%)
**total**	Total	**1472**	
	del(5q)		178
	UPD5q		8
	total 5q abnormality	**186**	(13%)

*Abbreviations*: CHART: continuous hyperfractionated accelerated radiotherapy; CFRT: conventional fractionated radiotherapy; SLR: sublobar resection; LF: local failure; RF: regional failure; LDF: local and distant failure; RDF: regional and distant failure; DF: distant failure; LRF: locoregional failure; WR: wedge resection; BT: brachytherapy; mths: months; 3D-CRT: three-dimensional conformal radiotherapy; NA: not reported.

MDS, myelodysplastic syndromes; RCUD, refractory cytopenia with unilineage dysplasia; RCMD, refractory cytopenia with multilineage dysplasia; 5q-, MDS with isolated del(5q); MDS-U, MDS unclassifiable; RARS, refractory anemia with ring sideroblasts; RAEB, refractory anemia with excess blasts; MDS/MPN, myelodysplastic/myeloproliferative neoplasms, including CMML, JMML, MDS/MPN-U, RARS-T, and atypical CML; MPN, myeloproliferative neoplasms, including MPN, idiopathic MF, systemic mastocytosis, essential thrombocythemia, CML, and polycytemia vera ; pAML, primary acute myeloid leukemia; sAML, secondary acute myeloid leukemia, includes therapy related myeloid malignancies.

**Figure 2:**
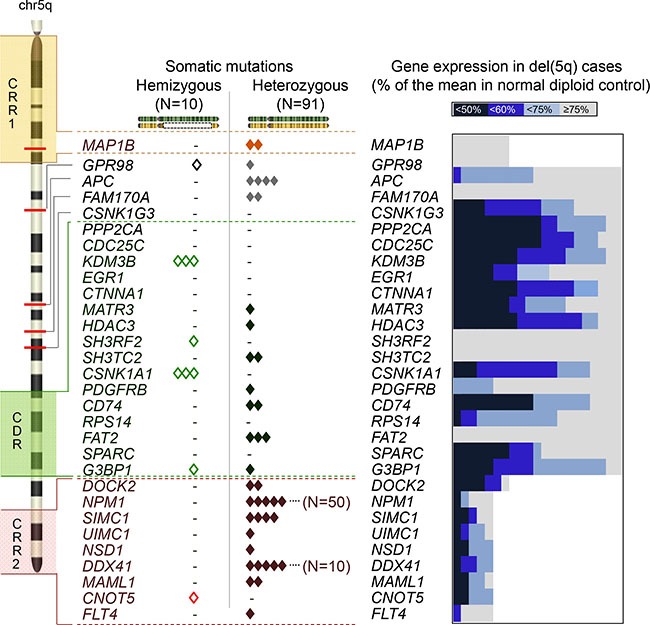
Zygosity of mutations and haploinsufficiency of the genes on chr5 An ideogram of chr5 in the left panel demonstrates 2 commonly retained regions (CRR1 and CRR2), a commonly deleted region (CDR) and the locations of the genes mutated in this study or previously reported to be pathologically important. Each somatic mutation was shown according to the configuration of zygosity; hemizygous (open diamonds) and heterozygous mutations (solid diamonds). In the right panel, the ratio (%) of the gene expression in each case with del(5q) (*N* = 5–21) was calculated as divided by the mean value in normal diploid chr5q samples (*N* = 17–162). The ratios in individual del(5q) cases were shown by gradient blue and gray plots as indicated in the figure. Decreased expressions less than 60% were considered haploinsufficient.

Our investigations focused on mutational events in 5q genes in both diploid and 5q deletion cases; 329 non-del(5q) cases and 60 cases of del(5q), including 13 low-risk MDS, 8 high-risk MDS, 14 sAML, 4 MDS/MPN(3 CMML and 1 MDS/MPN-U) and 21 pAML. In addition, 8 selected genes on chr5 (*e.g*., *DHX29*, *GPR98, APC, CSNK1A1, CSF1R, NPM1, SIMC1 and DDX41*), found to be mutated in WES cohort studies were investigated in other cases (*N* = 627) by targeted sequencing (Supplementary Table S1). We identified 583 non-silent alterations on chr5 (6% of whole exome alterations) predominantly located on 5q (505 alterations/275 genes). After stringent filtering to avoid false positives [18], we narrowed the focus of our investigations to “tier 1” mutations. All candidate alterations were validated by Sanger sequencing and/or targeted deep sequencing of DNA from both neoplastic and germ line DNA. In the entire cohort we identified 257 somatic mutations in 159 genes through 5q; present in 26% (16/60) of del(5q) cases and in 40% (131/327) of 5q diploid cases (Figure 1B–1C), including well-known *NPM1* mutations (*N* = 50 mutations), but also the newly identified recurrently mutated genes *CSNK1A1*, *G3BP1* and *DDX41* (Figure 2). No homozygous mutations were found within the long arm of chr5 in UPD cases.

When we focused on mutations in the CDR, we found 70 alterations in 57 genes (27% of all alterations on 5q) including *CSNK1A1* (5q32) and novel recurrently mutated genes *G3BP1* (*N* = 2, 5q33.1). Hemizygous alterations in most of cases were found in the CDR (*N* = 8, Figure 1C, Figure 2). Mutations of *CSNK1A1* (3/131 del(5q); 0/429 diploid cases) and *KDM3B* (3/60 del(5q); 0/331 diploid cases) were detected only in cases with del(5q). Mutations were found outside of the CDR, including *APC* (*N* = 4), *FAM170A* (*N* = 2) and *GPR98* (*N* = 2), the latter previously found to be involved in germ line mutation in Usher syndrome. In serially studied specimens a *GPR98* mutation appeared initially in a heterozygous configuration and was found later to be hemizygous upon evolution of del(5q). No mutations in *CDC25C*, *CTNNA1*, *RPS14, PPP2CA* and *SPARC* were identified. In genes corresponding to CRR1 we found 29 heterozygous alterations, including *MAP1B*. We also detected 96 alterations in CRR2, including *NPM1* (*N* = 50), *SIMC1* (*N* = 4), *DDX41* (*N* = 10) and *FLT4* (*N* = 1); these mutations occurred only in a heterozygous configuration.

### Expression of frequently mutated genes on chr5

Haploinsufficiency caused by deletion of 5q involving multiple genes is likely the key pathogenetic mechanism in 5q- syndrome. We hypothesized that heterozygous hypomorphic mutations ultimately result in haploinsufficient expression, thereby phenocopying haploinsufficiency due to deletions. We investigated the expression levels of genes located on 5q in comparison between cases with/without del(5q) focusing on genes which have been previously reported or we have found affected by heterozygous mutations. For definition of haploinsufficiency we set the cut-off value < 60% of normal. In total, 12/27 genes showed haploinsufficient expression in del(5q), with the majority of haploinsufficient genes located in the CDR (7 genes) or CRR2 (3 genes) (Figure 2). Within the CDR, *G3BP1*, *CD74* and *CSNK1A1* exhibited both haploinsufficiency and somatic mutations, whereas *PPP2CA, CTNNA1* and *CDC25C* showed haploinsufficient expression but no mutations. *SH3RH2* and *SH3TC2* genes did not display haploinsufficiency, while somatic events of these genes were noted. Other recurrently mutated genes located outside of the CDR: such as *APC*, *DDX41* and *MAML1* also showed haploinsufficient expression; however, mRNA levels of *GPR98*, *FAM170A*, a cluster of protocadherin family genes and *NPM1* were not decreased in deletion cases.

### Del(5q) and genetic events on other chromosomes

**Figure 3:**
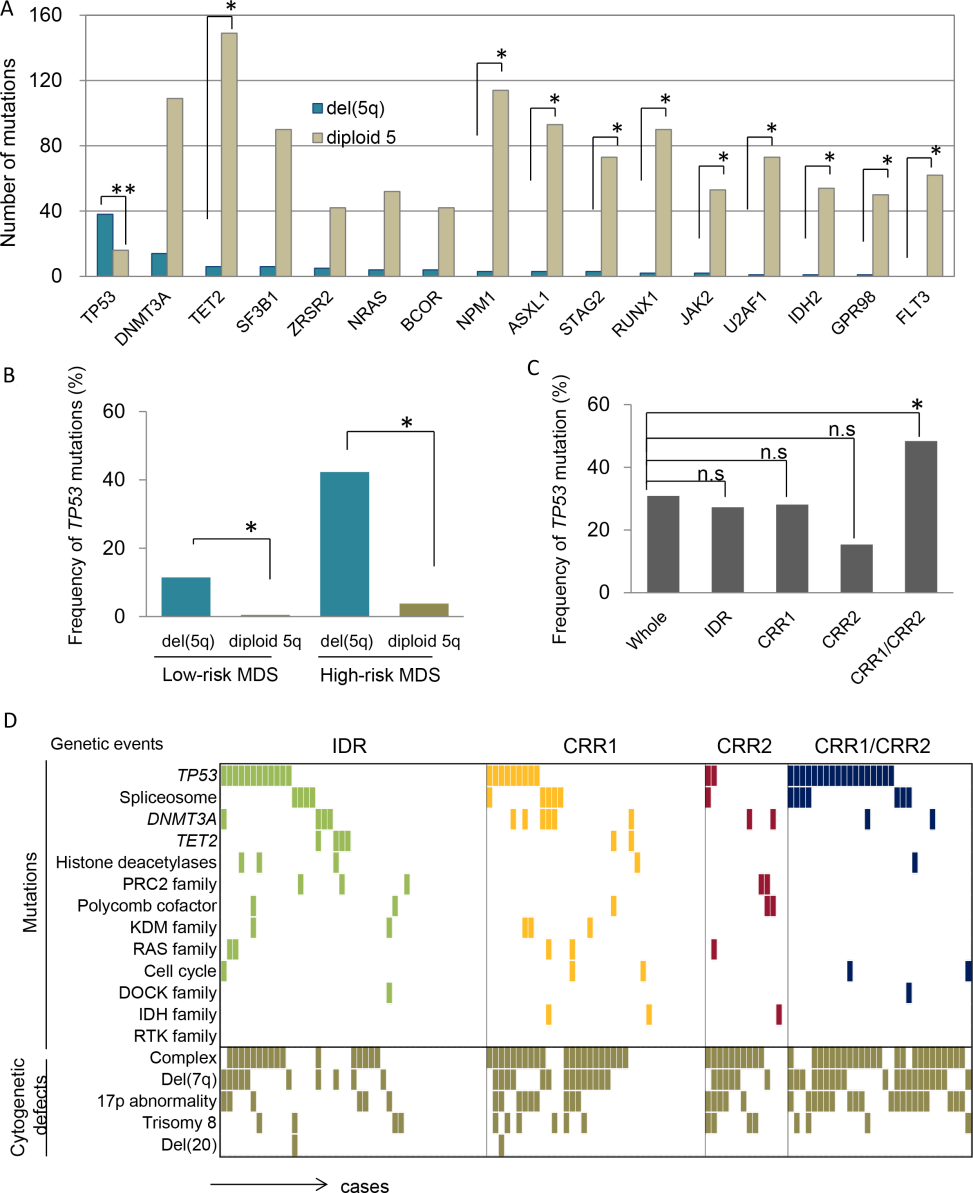
Presence of coexisting genetic events with del(5q) (**A**) In total, 1020 cases were analyzed for mutational landscape using NGS. Among them, 369 cases were MDS, 148 cases were MDS/MPN, 56 cases MPN, 146 cases were secondary-AML and 301 cases were primary-AML. Number of mutations were compared between cases with and without del(5q) (*N* = 894 and 126, respectively) *P < .005, **P < .0001. (**B**) Frequencies of *TP53* mutations were compared between del(5q) and diploid chr5q cohorts (Low-risk MDS; RCUD, RCMD, 5q-synd, MDS-U and RARS, High-risk MDS; RAEB-I and RAEB-II), *P < .001. (**C**) Frequencies of *TP53* mutations coexisting with subsets of del(5q) (as below) were individually compared to that with whole del(5q). IDR, interstitial deleted region (affecting q14.2-q34); CRR1, commonly retained region 1 (q11.1-q14.2); CRR2, commonly retained region 2 (q34-qter); CRR1/CRR2, deleted region spanning from CRR1 to CRR2, *P < .001. (**D**) Mutational spectrum associated with subsets of del(5q) were demonstrated according to the functionally relevant grouping of genes frequently mutated in myeloid neoplasms. Positive genes in each pathway were as follows: spliceosome, *SF3B1*, *LUC7L2* and *PRPF8*; KDM family, *KDM3B* and *KDM6B*; Ras family, *KRAS*, *NRAS* and *RIT1*.

The associated mutational landscape outside of the del(5q) region may also affect the clinical and biological features of del(5q) cases. We thus analyzed the potential relationship of somatic mutations observed in other chromosomes in del(5q) cases. Globally, 5q- syndrome cases were associated with lower numbers of mutations (average 2.5 mutations/case) compared to IDR deletions (9.5 mutations) and patients with extreme deletions (involving CRR1 and CRR2; on average 18 mutations) by WES. *TP53* mutations were associated with del(5q) as previously described by our group [19] and others [20–22]. In contrast, 10/15 of top mutated genes showed a significantly mutual exclusivity with del(5q) (e.g., *TET2*, *NPM1*, *FLT3-ITD/TKD*) (Figure 3A). Mutations of *DNMT3A*, *SF3B1*, *ZRSR2, NRAS* and *BCOR* were evenly distributed. The correlation of *TP53* mutations with del(5q) was most prominent. *TP53* mutations with del(5q) was mostly occurred with other chromosome abnormality, though, only 1 case was seen with isolated del(5q) (Supplementary Table S2). In low risk MDS, *TP53* mutations were detected in 13% of del(5q) cases and in only 0.5% of diploid 5q cases (Figure 3B; *P* = .0001). Among high-risk MDS, *TP53* was mutated in 42% of del(5q) *vs*. 4% of diploid 5q patients (Figure 3B; *P* < .0001). When we focused on the extent of the deletion, somatic *TP53* mutations were particularly frequent (39%, 17/38) in cases whose deletion involved both CRR1 and CRR2, and in 32% (12/38) of interstitial deletion cases (Figure 3C, P = .03). Moreover, large deletions tended to be a part of complex karyotypes and 17p abnormality (Figure 3D).

### Prognostic impact

**Figure 4:**
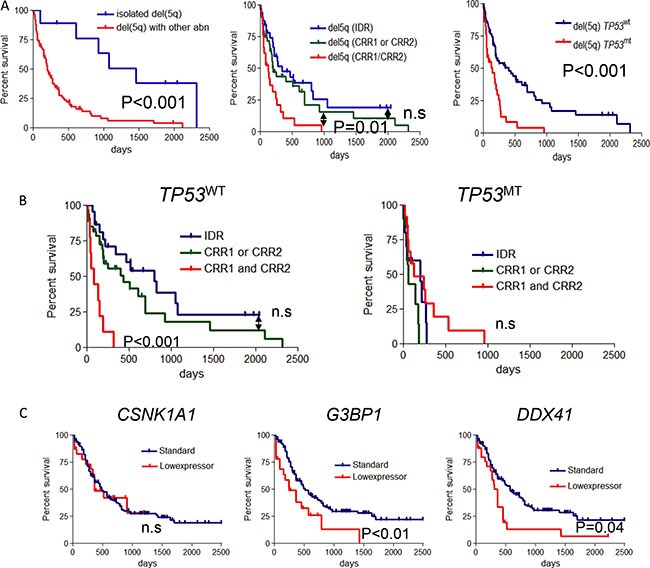
Survival analysis in the cases with del(5q) (**A**) (Left panel) Overall survival (OS) for patients with isolated del(5q) was compared among the del(5q) with other abnormality(del(5q) with abn). (Middle panel) Overall survival (OS) for patients with del(5q) was compared among the subgroups of 3 deleted regions as mentioned in Figure 1C. (Right panel) OS for patients with del(5q) were compared according to the *TP53* mutational status. *TP53*MT, *TP53* mutation; *TP53*WT, *TP53* wild type. (**B**) OS was compared in 3 groups of each deleted region as above in the cohorts of *TP53*WT (left) and *TP53*MT (right). (**C**) OS was compared between low (under mean-1.5 S.D. in all cohort) and normal-range expression of 3 genes in each panel (*CSNK1A1*, *G3BP1* and *DDX41*).

To investigate clinical implications, we initially assessed the impact of the deleted lesion on clinical outcomes, in which follow-up data were available. As expected, patients with isolated del(5q) showed better prognosis compared with del(5q) with other chromosomal abnormality (*P* < .001, Figure 4A). When we investigated the size of deletion, patients with both CRR1/CRR2 lesions (involving the 5q extremes) showed a worse prognosis compared with cases including CRR1 or CRR2, or with IDR lesions (*P* = .01, Figure 4A). We also investigated the impact of the presence of *TP53* mutations (*TP53*MT) as the most common mutational event associated with del(5q). Predictably, survival among patients with del(5q) was inferior for *TP53*MT compared to wild type *TP53* (*TP53*WT) cases (*P* < .001, Figure 4A). Furthermore, there were significant survival differences reflective of the previously described differences in the extent of deleted regions on 5q in *TP53*WT cases (*P* < .001, Figure 4B). *TP53*MT cases showed inferior outcome regardless of deleted region (Figure 4B).

When we focused on the prognostic value of low-expressed genes in primary AML cohort, low expression of *G3BP1* and *DDX41* correlated with a shorter survival (*P* < .001 and *P* = .04, Figure 4C), an effect that was not seen in *CSNK1A1* cases (Figure 4C). We also could not find association between low-expression and outcome in *MAP1B, GPR98* and *FAM170A* which did not reach the haploinsufficiency cut off in our cohort (data not shown).

### Del(5q) and hierarchical clonal architecture

**Figure 5:**
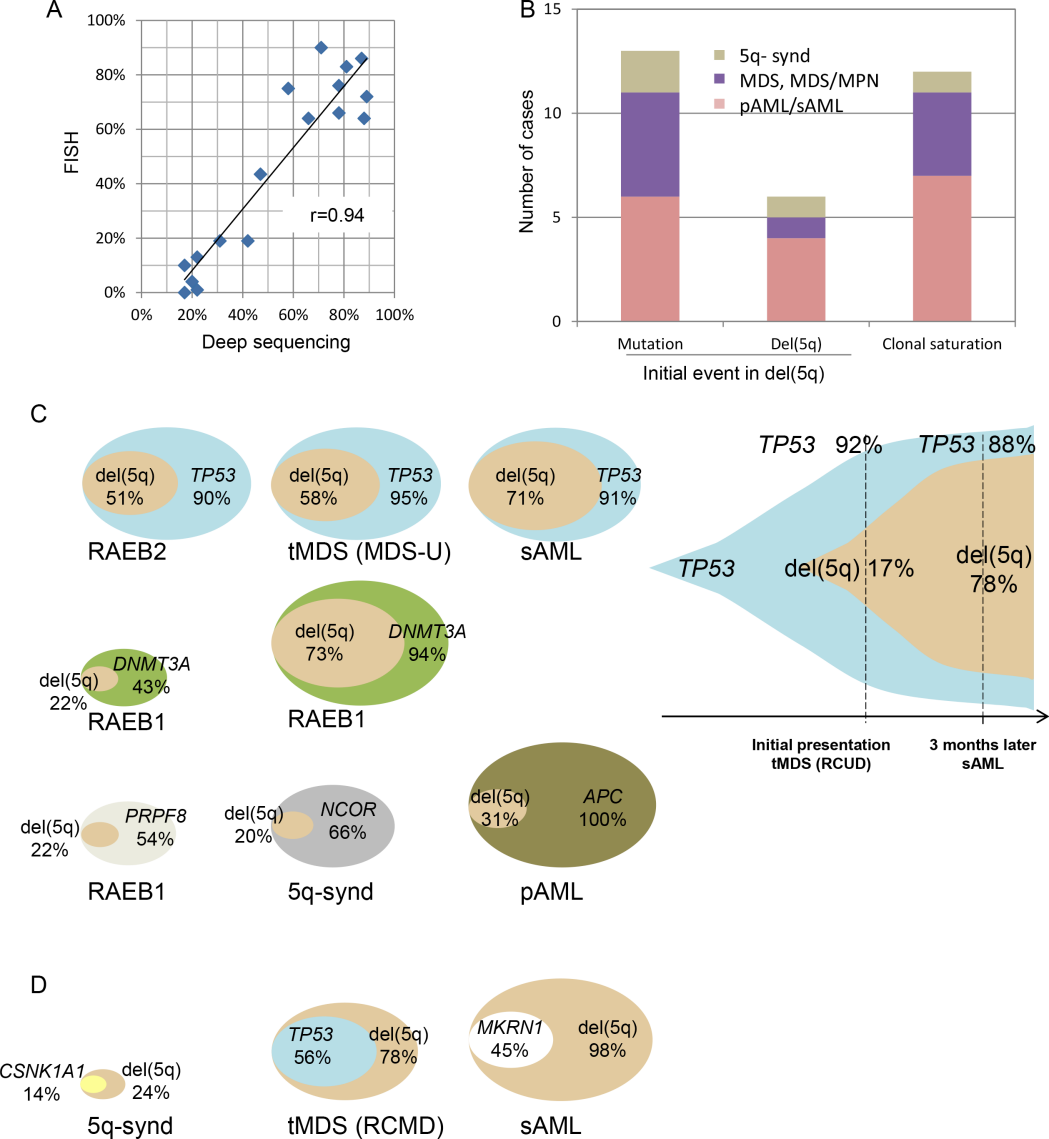
Ancestral mutations and subsequent del(5q) clones (**A**) Correlation between FISH and deep sequencing on the detection of del(5q) clone. (**B**) Initial genetic events (mutations or del(5q)) were determined by the size of affected clonal cell populations based on the following assessments (bar graph). Variant allelic frequencies (VAF) of somatic mutations were adjusted by copy numbers and LOH was based on SNP-A results. Pathogenic genes mutated as initial events included *TP53*, *DNMT3A*, *NCOR2* and *PRPF8* as shown in Figure 5C. VAF of SNPs with a deleted-allele due to del(5q) were calculated for allelic imbalance as mentioned in the methods section. Inconclusive cases without discrimination were categorized as clonal saturation. Distribution of the disease phenotypes in each clonal pattern was demonstrated by colors as indicated. (**C**) Clonal architecture of the cases (*N* = 9) with initial driver mutations prior to del(5q) was demonstrated by overlaid double-oval figures (left) and a serial-assessment figure (right). Percentages indicate the fraction of the cells affected by mutations or del(5q). (**D**) In 3 other cases, del(5q) was shown to be a primary event.

It has been presumed that the del(5q) is the ancestral event in the myeloid neoplasms harboring this lesion [23]. Using deep sequencing and ’allelic imbalance’ we can determine the position of del(5q) in the hierarchical clonal architecture [24]. In our cohort, del(5q) was present in 17–98% of tumor cells and there was good correlation to the size of del(5q) clone by FISH (*r* = .94; Figure 5A). We identified three patterns of recurrent clonal architecture in del(5q) cases (Figure 5B) i) apparent pathogenic somatic mutations precede the deletion event (31%), ii) del(5q) appears to precede any other somatic mutation (19%) and iii) the succession cannot be determined because of expanded clones with similar size (“clonal saturation”) *i.e*., these cases were not informative. In our cohort, among the majority of cases in which del(5q) was a secondary lesion, in 64% of instances a *TP53* mutation was the ancestral event and in 27% of cases the primordial lesion was *DNMT3A* mutation (Figure 5C). When we compared different time points in same case (MDS-phase and leukemic phase), the proportion of cells affected by *TP53* mutation was more prominent than that of del(5q) (92% vs. 17%, Figure 5C right). In contrast, *CSNK1A1* mutation occurred in the remaining allele after del(5q) (Figure 5D). We also detected 1 case in which del(5q) was asserted to be ancestral, and the *TP53* mutation was detected as a secondary event.

## DISCUSSION

Our study identified a cohort of 178 patients with various forms of del(5q) to answer several fundamental questions related to the pathogenesis of myeloid malignancies associated with these deletions: i) is del(5q) associated with recurrent hemizygous mutations; ii) may heterozygous mutations corresponding to haploinsufficient genes mimic the phenotype of the deletion; iii) are gene mutations on other chromosomes recurrent in del(5q); iv) what is the architecture and clonal evolution pattern in del(5q) myeloid neoplasms? Does 5q still stand as the primordial lesion in the light of data generated from the use of the new genomic platforms?

We found several somatic hemizygous mutations in del(5q) cases, including *G3BP1* and *CSNK1A1*. Mutations in *CSNK1A1* were found in a canonical E98 position as recently reported [16, 25]; only hemizygous mutations were found with del(5q) in the context of various clinical subtypes, including aggressive diseases RAEB-1 or therapy-related MDS. *CSNK1A1* E98 mutations increase β-catenin activity thereby providing selective growth advantage. *G3BP1* is another gene encoded within the CDR, and unlike *CSNK1A1* mutations those in *G3BP1* occurred both in heterozygous and hemizygous configuration. G3BP1 is known to control p53 activity through a dual pathway involving direct protein interaction of G3BP1-p53 and deubiquitination by regulating the ubiquitine specific peptidase USP10 [26].

Several other hematopoiesis-related genes and tumor suppressor genes are located in the CDR (*e.g*., *CTNNA1*, *PPP2CA*, *EGR1*, *SPARC*, *RPS14* and *CDC25C*) with previously reported [14, 27–31], however, we were unable to detect hemi- or heterozygous mutations in these genes. It is possible that they contribute to clinical heterogeneity, shape the clinical phenotype or modulate the growth advantage of the del(5q). For the purpose of our investigations we hypothesized that haploinsufficient genes in del(5q) may also be affected by loss of function/hypomorphic mutations in diploid cases, and we have identified several genes fitting this profile. They were affected only in a minority of patients, and most did not recapitulate the clinical features of del(5q). Of note is that even the haploinsufficient expression showed variability among del(5q) cases: while average expression values may be decreased in del(5q) cohorts, specific expression is indeed haploinsufficient only in a portion of cases, and may in part reflect the relative percentage of the malignant clone in each patient. These differences in the degree of haploinsufficiency may explain, in addition to the size of deleted region, the intrinsic diversity of del(5q). Epigenetic regulation also affects the expression of each genes on del(5q) whereby deletion of unsilenced allele could even lead to gain of silencing. However, there would be no impact if silenced allele is deleted and thus may not be a key determining factor for the degree of haploinsufficiency. Because del(5q) occurred in one allele but epigenetic regulation (hyper- or hypo methylation) occurred in both allele, most likely at random. Nevertheless, several genes were found to be haploinsufficient and affected by somatic mutations, including *HDAC3*, *CSNK1A1*, *G3BP1* and *DDX41*. Moreover, the functional role of 5q genes in hematopoiesis has been shown using a murine model.

The number of somatic mutations on other chromosomes increased with the increasing length of the 5q deletion. Co-occurrence of a *TP53* mutation was particularly prominent in this del(5q) cohort, as reported in other studies [19, 21, 22]. It is still unclear why *TP53* mutations selectively coincide with del(5q), one could speculate that loss of p53 function might overcome p53 tumor suppressor effects and foster leukemia evolution. Of note is that there are several gene clusters of negative regulators of *TP53* on chr5q, such as, *PPP2CA*, *RPS14*, *CSNK1A1* and *G3BP1*.

Among del(5q) patients we found that inferior survival was associated with patients with both deletion of CRR1 and CRR2. This relationship became evident in the *TP53*-wild type cohort, whereas there was no survival difference in existence of *TP53* mutation. The larger deletions were frequently associated with other chromosomal abnormalities, which associated with inferior survival [32, 33]. However, the reasons are still unclear. One possibility is that there are several tumor suppressor genes in this location and long deletion causes multifunctional loss of these genes. Alternatively, other gene mutations or loss of function of tumor suppressor gene may result in different clinical phenotype of extended version of del(5q). We also analyzed impact of a cohort of individual genes with low expression, which indicated recurrent somatic mutations. Low expression of *G3BP1* or *DDX41* correlated with inferior survival but there was no prognostic impact in *CSNK1A1*, *CDC25C* and *EGR1* (data not shown). These results indicate that the presence of low expression did not always correlate with survival, and the loss of tumorsupressive function may affect their outcome. Thus, a loss of tumor suppressive function of *G3BP1* or *DDX41* may lead to leukemic evolution in del(5q).

To define the position of del(5q) within the clonal hierarchy, we have compared the clonal size of somatic mutations with that of del(5q) using a novel approach focusing on allelic imbalance. While it has been reported that del(5q) occurs in stem cells as an ancestral event in patients with the 5q- syndrome [23], our results indicate that the mutation of *TP53* or other driver gene mutations such as *DNMT3A* occurred as initial events followed by deletion of 5q in a majority of case. Previously, co-occurrence of a *TP53* mutation was described in various del(5q) cohorts [19, 21, 22], but the position of del(5q) and *TP53* mutation within subclonal hierarchy could not be precisely established using Sanger sequencing. In this report we were able to overcome this shortcoming using NGS. We also found somatic mutation of *APC* (5q22) as initial event in del(5q) case, a role for low expression of *APC* in the pathogenesis of myeloid neoplasms [34].

In summary, comprehensive molecular analyses using SNP-A karyotyping, WES and targeted sequencing revealed recurrent somatic mutations involving *CSNK1A1* and *G3BP1* in the CDR and *DDX41* in the CRR in myeloid neoplasms with the del(5q). These genes showed haploinsufficiency in deleted cases and low expression of *G3BP1* or *DDX41* is associated with poor survival, which may be due to loss of function. In addition, in assessing allelic imbalance in del(5q), our results suggested that del(5q) is not an universal ancestral event. Mutation of *TP53* is the most common mutation in del(5q) cases and may serve as an ancestral event. These data illuminate the impact of the del(5q) in myeloid malignancy, providing deep insights into the identity and role of key genes.

## MATERIALS AND METHODS

### Samples

Paired bone marrow and germ line (GL, CD3^+^ lymphocytes) DNA was obtained from 389 patients with various myeloid neoplasms and additional 631 DNA samples were included for further targeted resequencing, including a total of 178 cases of -5/del(5q) (Table 1). All samples were obtained following written informed consent approved by the institutional review boards at Cleveland Clinic and the University of Tokyo. The Cancer Genome Atlas (TCGA) AML data set was obtained from http://cancergenome.nih.gov/.

### Sequencing, SNP array analysis and gene expression

Single-nucleotide polymorphisms (SNP) array analysis and WES was performed by HiSeq 2000 (Illumina) and result analyzed as previously described [5]. All the selected observations were validated by targeted Sanger sequencing or PCR-amplified deep sequencing using MySeq (Illumina). Targeted sequencing was performed by TruSeq custom amplicon (Illumina). Previously published microarray expression data were obtained on a cohort of 183 MDS patients (-5/del(5q), *N* = 41) and healthy controls (*N* = 17) (Supplementary Figure S1) [5, 17].

### Determination of clonal burden

The detection of clonal size of del(5q) was accomplished by calculation of allelic imbalance for informative SNPs present within deleted region in heterozygous configuration in GL. For all heterozygous SNPs in the region, label the lost allele *A* and the retained allele *B*. For reads covering a heterozygous site in the sample, the probability that the read will carry the *B* allele is: *P*[reading *B* allele] = *P*[reading *B* allele | read from 5q^−^] × *P*[read from 5q^−^]+P[reading *B* allele/read from non-5q^−^] × P[read from non-5q^−^]. The terms in this expression depend on clonal abundance *c* of the deletion, and may be computed as: *P*[reading *B* allele/read from 5q^−^] = 1, *P*[reading *B* allele/read from non-5q] = 0.5, *P*[read from 5q^−^] = *c*/(2 × (1–*c)*+*c*)=*c*/(2–*c*), *P*[read from non- 5q^−^] = 2 × (1–*c*)/(2 × (1–*c*)+*c*)=(2–2*c*)/(2–*c*). Substituting these values into the expression and algebraically simplifying gives the expected proportion of reads harboring the *B* allele as *p* = 1/(2–*c*). Solving for *c*, the proportion for cells harboring the deletion is approximately *c*≈2–(1/*p*). To combine information from all *n* (GL) heterozygous SNPs in the region, we estimated *p* across these SNPs as, where the allele *B*i for each SNPi is that with the highest read frequency.

### Statistical analysis

Comparisons of proportions and ranks of variables between groups were performed by the χ^2^ test, Fisher exact test, Student *t* test or Mann-Whitney *U* test, as appropriate. We used the Kaplan-Meier and the Cox method to analyze overall survival (OS) with a 2-sided *P* less than or equal to .05 determining significance.

## SUPPLEMENTARY MATERIALS FIGURES AND TABLES






