# Supplementary material for: Recurrent genetic defects on chromosome 5q in myeloid neoplasms
Source: Oncotarget. 2016 Dec 23;8(4):6483–95. doi: 10.18632/oncotarget.14130 (PMC5351647; doi:10.18632/oncotarget.14130)
Supplement: Supplementary file 1 [file oncotarget-08-6483-s001.pdf]

Recurrent genetic defects on chromosome 5q in myeloid neoplasms

Supplementary Materials

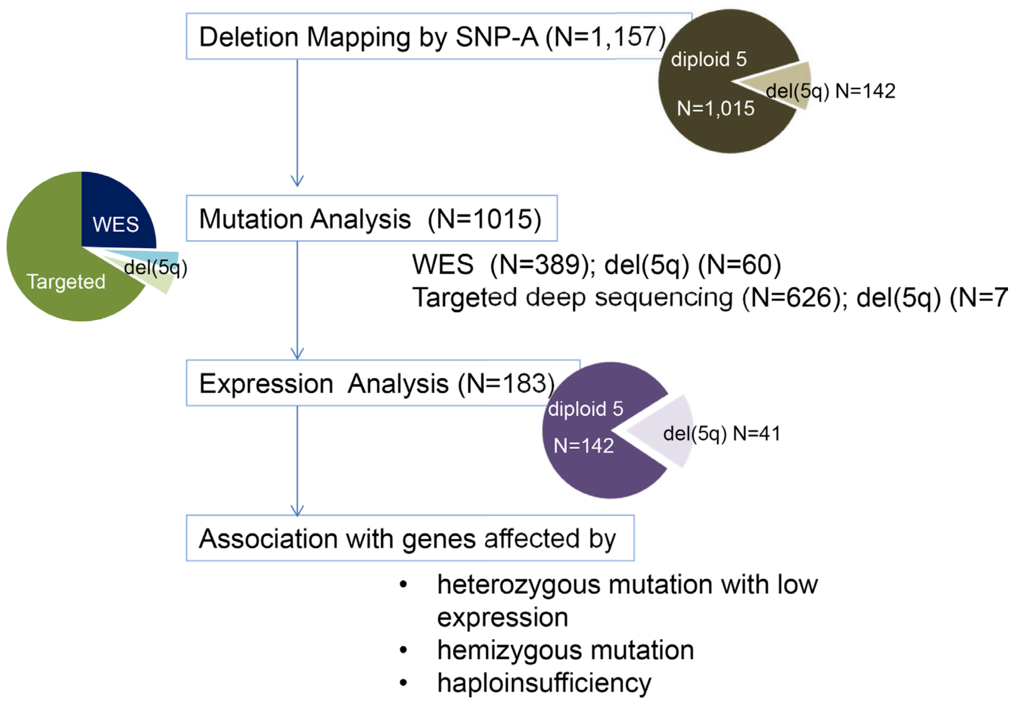

Supplementary Figure S1: Detection of mutational events in 5q genes. To detect putative pathogenic genes on chromosome 5q we performed a comprehensive analysis using SNP-array, whole exome sequencing, targeted deep sequencing and expression arrays. In addition, we assessed the location of del(5q), the zygosity of mutations, haploinsufficiency, the frequency of each defects, and the associations with such individual factors.

Supplementary Table S1: Clinical characteristics of the targeted sequence cohort

	total cohort	del(5q)
**Number**	626	73
Age at diagnosis (years±s.d.)	67 (± 13.9)	65 (± 13.8)
range	18–100	21−88
**Gender**		
Male:Female	1:0.62	1:1.09
**Disease at sampling**		
MDS-Low	166 (26.5%)	29 (40%)
MDS-High	98 (15.6%)	18 (25%)
MDS/MPN	105 (16.8%)	2 (2.7%)
MPN	50 (8.0%)	0
pAML	96 (15.3%)	9 (12.3%)
sAML	111 (17.7%)	15 (20.5%)
**Cytogenetics**		
Normal	255 (40.7%)	
del(5q)	73 (11.7%)	
−7/del(7q)	48 (7.7%)	4 (5.6%)
Complex (≧ 3)	25 (40.0%)	38 (52.8%)
trisomy 8	40 (6.4%)	2 (2.8%)
other	124 (19.8%)	10 (13.9%)
Not Available	61 (9.7%)	9 (12.5%)

MDS-Low, includes RCUD, refractory cytopenia with unilineage dysplasia; RCMD, refractory cytopenia with multilineage dysplasia; 5q-, MDS with isolated del(5q); MDS-U, MDS unclassifiable and RARS, refractory anemia with ring sideroblasts. MDS-High includes RAEB, refractory anemia with excess blasts. MDS/MPN, MDS/myeloproliferative neoplasms;MPN myeloproliferative neoplasms; pAML, primary acute myeloid leukemia; sAML, secondary acute myeloid leukemia, includes therapy related myeloid malignancies.

Supplementary Table S2: Cytogenetic and TP53 mutations in patients with del(5q). See_Supplementary_Table S2

Supplementary Table S3: Mutations on chr5. See_Supplementary_Table S3
