# Supplementary material for: Recurrent genetic defects on chromosome 5q in myeloid neoplasms
Source: Oncotarget. 2016 Dec 23;8(4):6483–95. doi: 10.18632/oncotarget.14130 (PMC5351647; doi:10.18632/oncotarget.14130)
Supplement: Supplementary file 2 [file oncotarget-08-6483-s002.docx]

**Supplementary Table 2.**Cytogenetic and TP53 mutations in patients with del(5q).

|  | Diagnosis | Cytogenetics | TP53 |
| --- | --- | --- | --- |
| Low risk MDS | RCUD | 44-47,XX,del(5)(q12q33)[11],-7[9],del(7)(q22)[2],del(7)(q22q34)x3[1],-8,-12,-16,-16,-17,-18[10],+21[8],+1-4r[12],+mar1,+mar2[11],+mar3[10],+mar4[10][cp12]/46,XX[8] | MUT |
|  | RCMD | 41-44,XY,del(1)(p21)[1],del(3)(q21q26.2)[2], inv(3)(q21q26.2),add(5)(q22),-6,add(7)(q22),-10[19],add(11)(q23)[3],-12,-17,+mar1[19],+mar2[18][cp20] | MUT |
|  | RCMD | 46,XY,add(5)(q11.2),add(8)(q24.1) | MUT |
|  | RCMD | 44,XY,del(2)(q31),add(5)q35),?inv(5)(p15q31),-6,-7,-8,-18,+mar2,+mar3[4]/46,XY[16] | MUT |
|  | RCMD | 48-53,XY,-4,del(5)+13,+19,+21,+22,+2r,+1-4mar[cp19]/46,xy[1] | MUT |
|  | 5q-synd | 46,XX,del(5)(q12q33)[cp7] | WT |
|  | 5q-synd | 46,XX,del(5)(q13q33)[19]/46,XX[1] | WT |
|  | 5q-synd | 46,XX[20]. | WT |
|  | 5q-synd | 46,XY,del(5)(q12q33)[6] | WT |
|  | 5q-synd | 46,XX,add(5)(q13)[20] | WT |
|  | 5q-synd | 46,XX,del(5)(q13;q33) [15],46,t(1;2)(p31;p13)[3] | WT |
|  | 5q-synd | 46,XX,del(5)(q13q33)[17]/46,XX[3] | WT |
|  | 5q-synd | 46,XX,del(5)(q13q33)[19]/46,XX[1] | WT |
|  | 5q-synd | 46,XX[20] | WT |
|  | 5q-synd | 46,XX,del(5)(q14q32)[16]/46,XX[4] | WT |
|  | MDS-U | 46,XX[20] | WT |
|  | MDS-U | 47,XX,+8[2]/47,idem,del(5)(q22q33)[2]/46,XX[17] | WT |
|  | RCUD | 45,XY,t(1;4)(q32;q35),del(5)(q13q33),del(8)(p11.2),-21[20] | WT |
|  | RCUD | 46,XY[20] | WT |
|  | RCUD | 46,XX,del(5)(q13q33)[13]/46,xx[7] | WT |
|  | RCMD | 45,XY,del(5)(q13q33),-7,-17,-18,+mar1,+mar2[20] | WT |
|  | RCMD | 45,X,-X[20] | WT |
|  | RCMD | 45,XX,del(5)(q15q33),del(7)(q22),-22[cp2]/46,XX[ | WT |
|  | RCMD | 46,XX,del(5)(q15q33)[3]/46,XX[17] | WT |
|  | RCMD | 46,XX,ider(20)(q10)del(20)(q11.2q13.3) | WT |
|  | RCMD | 46,XX,t(2;11)(p23;q23),del(5)(q2?2q3?4)[19]/46,XX[1] | WT |
|  | RCMD | 46,XY[20] | WT |
|  | RARS | 44-48,XY,+Y[2],-5[4],del(5)(q13q31)[9],-7[3],del(7)(q22)[2],+8[5],del(11)(q23)[9],add(12)(p11.2)[11],+19[2],-20[5],+mar1[10],+1-5mar[7][cp13] | WT |
|  | RARS | 49-53,XY,+1,add(1)(p32),-4,del(5)(q22q35),+6,der(7)t(1;7)(p32;q32),+8,+9,+1 | WT |
|  | RARS | 47,XY,+8[cp18]/46,XY[2] | WT |
|  | RARS | 48,XY,del(5)(q12q33),-6,del(7)q11),+8,-13,i(17)(q10),+3mar[4]/46,XY[3] | WT |
| High risk MDS | RAEB-1 | 44-50,XY,add(3)(p12)[17],　+add(3)(p12)[13],del(5)(q14)[18],dic(6;22)(p11.2;q13)[16],+dic(6;22)(p11.2;q13)[4],+22[17],+mar1[4],+mar2[3],+mar3[3][cp18]/46,XY[2] | MUT |
|  | RAEB-2 | 41-43,XY, add(1)(p32), add(5)(q11.2), -7, add(12)(p13), -13, -17, -18, -20, +1-2 mar(cp19)/46,XY (1). | MUT |
|  | RAEB-2 | 43,XX,-3,del(5)(q22q35),-7,add(12)(p13),-16[4]/43-44,idem,-2,add(18)(p11.3),+mar1,+mar2[cp15]/ 46,XX[1] | MUT |
|  | RAEB-2 | 44,XX,add(3)(q21),-5,t(7;12)(q22;p13),-8,der(16)t(8;16)(q13;q11.2)[19]/46,XX[1] | MUT |
|  | RAEB-2 | 46-47,XY,add(3)(q11.2),del(5)(q13q33),+8,-15,-16,+1-2mar[20] | MUT |
|  | RAEB-2 | 44-47,XY,-4[12],-5[5],del(5)(q13q33)[15],-7[9],add(7)(p15)[5],add (7)(q36)[4],+8[10],del(12)(p11.2)[5],der(19)t(4;19)(q12;q13)[5], -20[20],+r[2],+1-3mar[15],+1-2f[7][cp20] | MUT |
|  | RAEB-2 | 45,XY,add(2)(q33),-5,del(5)(q15q33),del(9)(q12q22)[3],-10,del(11)(q23) ,del(12)(q13),-14,-15,-17,+4mar][cp12]/44,idem,del(3)(q22),add(4)(q35) ,del(7)(q22)[7]/46,XY[1] | MUT |
|  | RAEB-2 | 46,XX,-3,del(5),-16,add(17),+20,+1-3mar | MUT |
|  | RAEB-1 | 43-44,XY,del(5)(q13q35),-7,add(9)(p24),psu dic (17;8)(p13;p23),-18,psu dic (19;14)(q13.4;p13),+1-2r[cp20] | WT |
|  | RAEB-1 | 46,XX,del(5)(q15q33)[2]/46,idem,t(2;12)(p13;p11.2)[7] | WT |
|  | RAEB-1 | 47,XX,del(5)(q22q35)[1]/47,sl,+9,add(16)(q24)[3]/46,XX[17] | WT |
|  | RAEB-1 | 41-47,XX,add(2)(p21),add(3)(q27),der(5)t(5;11)(q22;q13),-11, -17[19],i(17)(q10),-18[19],-20[19],add(21)(p11.2)[18], +0-5mar[cp20] | WT |
|  | RAEB-1 | 44-47,X,-Y,del(2)(p12),-3,-5,-6,del(6)(q12),-7,+8,-9,-11,-11,-12,add(17)(p12),+21,+1-6mar [cp12]/47,XY,del(6)(q12)[1]/45,X,-Y[1]/46,XY[5] | WT |
|  | RAEB-1 | 44-47,XX,der(2)t(2;4)(q21;q25),-4,del(5)(q22q35),+8,+8[1][cp6]/46,XX[9] | WT |
|  | RAEB-1 | 45,XX,add(3)(p21),-5,add(9)(q34),der(20)t(11;20)(q13;q13.3)[5]/45,sl,del(13)(q14q32)[10]/44,sl,-7,add(12)(p11.2),-18,+r[5] | WT |
|  | RAEB-1 | 45,XX,del(5)(q13q33),t(7;12)(q22;q13),t(13;22)(q34;q11.2),17[16]/45,XX,add(4)(p16),add(4)(q35),del(5)(q13q33),t(7;12)(q22;q13),del(11)(q23)(q34;q11.2),-15,-18,+2mar[1] | WT |
|  | RAEB-1 | 46,XY,del(5)(q15q33)[15]/46,XY[5] | WT |
|  | RAEB-1 | 47,XX,del(5)(q22q35),+9,add(16)(q24)[12] | WT |
|  | RAEB-2 | 42-47,X,-Y,-5,add(7)(p13),del(10)(q23q25),-13[16],add(13)(q34)[3],add(17)(p10),-18,del(19)(q13.1q13.3),-20,+mar1,+1-5mar[cp19]/46,XY[1] | WT |
|  | RAEB-2 | 43,XY,del(5)del(7)-12,del(13)add(16),-18,-21,-22,+mar1 | WT |
|  | RAEB-2 | 44,XY,del(5),-7,-18/43-45,idem,add(1)add(4),-22,+1-2mar[cp4]/46,xy[14] | WT |
|  | RAEB-2 | 46,XX,?add(7)(q22) or ?del(7)(q22q22)[5]/46,XX[15] | WT |
|  | RAEB-2 | 46,XY,del(2)(p11.2p15),del(5)(q13q33)[12] | WT |
|  | RAEB-2 | 70,XX,-X,-X,-1,-1,-1,-2,-2,-3,-3,-3,-3,-4,-5,-5,-5,-7,-7,-9,-11,-13,+14,+14,-15,-15,-17,-17,-17,-18,+19,+21,+21, -22[1]/46,XX[29] | WT |
|  | RAEB-2 | 46,XY,del(3)(q13.1q24)[21]/46,idem,inv(4)(p15.3q12)[3]/46,idem,del(12)(p11.2)[3]/46,XY[3] | WT |
| MDS/MPN | MDS/  MPN-U | 46,XY,del(5)(q12q33),del(13)(q12q14)[20] | MUT |
|  | MDS/  MPN-U | 46,XX,del(5)(q13q33)[4]/47,idem,+21[16] | WT |
|  | CMML-1 | 45,XX,der(1)del(1)(p34)add(1)(q42),del(5)(q13q33),-7[19]/46,XX[1] | WT |
|  | CMML-2 | 45,XY,-7[10]/46,XY,del(5)(q13q33)[1]/46,XY[9] | WT |
| pAML | pAML | 46,XY[20] | MUT |
|  | pAML | 46,XY,del(1)(p13p34),add(3)(q21),-5,del(12)(p11.2p13),-17,-19,+2,-5mar[9]/46,XY[11] | MUT |
|  | pAML | 52-55,XY,+Y, +4,+del(5)(q15q33),+6,+8,+add(10)(p11.2)x2,+11,add(11)(p15)x2,del(13)(q12q14),+14,+del(15)(q22q24),+17,+18,+21,+22[cp5]/　61-63,XY,+X,+Y,+1,+2,+4,+del(5)(q15q33),+6,+8,+8,+10,add(10)(p11.2)x2,+11,add(11)(p11.5)x2,+del(13)(q12q14),+14,+15,+del(15)(q22q24),+17,+18,+21,+22,+22,+mar[cp15] | MUT |
|  | pAML | 46,XX[20]. | WT |
|  | pAML | 46,XX,del(5)(q31q35)[13]/46,XX[7] | WT |
|  | pAML | 47,XX,del(5)(q12q33),+?del(12)(q13)[20] | WT |
|  | pAML | 41-53,XX,-X[5],der(7)t(7;2)(7;q21)[20],+5[3],der(5)t(5;17)(q13;q21)[20],add(6)(p21)[16],-7[20],-8[20],-11[20],-13[16],add(15)(p11,2)[3],-17[20],-21[19],+mar1[19],+mar1[3],+mar2[19],+mar3(8),+mar4[17],+mar5[10],+1-4mar[8][cp20] | WT |
|  | pAML | 42,XX,del(1)(q32),add(2)(q13),-4,-4,-5,del(6)(q13),der(7)t(1;7)(q23;q11.2),8[4],+9,add12(p13)[3],-15[8],-17,-18,-19,-20,+mar1,+mar3[6],+mar8[8],+mar[4][cp12]/47,XX+22[1]//46,XY[7] | WT |
|  | pAML | 43,XX,del(3)(p21),del(5)(q12q33),-9,add(11)(p15),-12,der(13)t(9;13)(p11;p11.2),-21[cp4]/42,sl,-16[7]/43,sdl1,del(2)(q21)[2]/41,XX,del(3)(p10),del(5)(q12q33),-7,-9,add(11)(p15),-12,add(13)(p10),der(13)t(9;13)(p11;p11.2),-14,add(17)(p13),-21[2]/42,idem,+mar1[3]/46,XX[2] | WT |
|  | pAML | 43,XY,del(5)(q13q33),der(15;21)(q10q10),-18,-19,add(20)(q13.3)[cp19]/46,xy[1] | WT |
|  | pAML | 46,XX,del(5)(q12q33)[4]/46,XX[16] | WT |
|  | pAML | 46,xy,del(3),add(4),add(5),del(5)del(11)add(12)+1-3mar[5][cp20] | WT |
|  | pAML | 47-52,XY,add (1) (p13),-5, dic (6;11) (p21;11.2), -7, add (8) (q24), -13, -16, -17,der (19) t(1;19) (p13;p13),+der (19) t(1;19)(p13;p13),add(22),(p11.2),+mar1,+mar2,+mar3,+mar4,+mar5,+mar6,+mar7,+1-5mar[cp19]/46,XY[1] | WT |
| sAML | sAML | 41-45,XX,-4,-5,der(6)add(6)(p21.3)add(6)(q23),inv(7)(p13q36),-10,ins(14;?)(q24;?),-15,add(17)(p11.2),add(19)(p13.3),der(22)t(15;22)(q15;q13),+1-4mar[cp19]/46,XX[1] | MUT |
|  | sAML | 42,XY,add(1)(q32),del(3)(q12),add(4)(q21),del(5) (q22q35),-7,der(12)t(12;13)(p13;q12),-13,-13,-16,-18,+mar1[cp20] | MUT |
|  | sAML | 44,XX,add(3)(p11),add(3)(q29),del(5)(q13q31),add(9)(q11),der(11)(11pter->11q1?3::hsr::14q11.2->14qter),hsr(11)(q23),-14,add(14)(p12),-19[cp19]/46,XX[1] | MUT |
|  | sAML | 45,XX,del(5)(q12q33),-7,+8,del(16)(q22),der(20)t(17;20)(q21;q13.3)[16]/46,idem,-13,-15,+mar1[4]. | MUT |
|  | sAML | 45,XY,del(5)(q13q34),del(7)(q22),+8,-17,-18[cp11]/46,XY[9] | MUT |
|  | sAML | 45-46,XX,add(4)(q21),-5[3],psu dic(5;?)(q11.1;?)[16], add(8)(p23),-13[17], -16,add(17)(p11.2),-18,+r[5],+mar1,+mar2[cp20] | MUT |
|  | sAML | 46,XX,del(5)(q13q33)[cp20]. | MUT |
|  | sAML | 42-46,XY,-3[17],der(5;17)(p10;q10)[16],add(7)(q22),-8[4],del(12)(p11.2p13)[14], der(13)t(11;13)(q13;q34)[6],+13[2],add(17)(p13)[4],-18[16],+22[2],+0-1r,+0-2mar[cp18]/88,idemx2[cp2] | MUT |
|  | sAML | 43-47,XX,add(1)(p36.3),der(3)t(3;11)(p11.2;q11)[3], der(3) add(3)(p21) add(3)(q21)[13],-4,-5,add(5)(q13),-7,-11, add(13)(p11.2)[2],-18,del(20)(q13.1),-21+2-7mar[5][cp17]/46,XX[3] | MUT |
|  | sAML | 44,X,-Y,del(5)(q22q35),-7,add(10)(q26),i(11)(q10),-16,-18,-20,+r,+mar1,+ace [cp16]/44,X,-Y,dup(5)(q13q35),del(8)(p23),-15,-20,+r,+ace[cp3]/46,XY[1] | MUT |
|  | sAML | 45,XX,del(5)(q15q33),del(7)(q22),-22[cp2]/46,XX[18] | MUT |
|  | RCMD | 45,XY,del(3)(p14),del(5)(q12q33),7,add(10)(p15)[14]/45,idem,del(12)(p11.2)[4]/　46,XY[7] | MUT |
|  | sAML | 46,XX,del(5)(q21q33),del(6)(p22),+9, add(9)(q11)x2,-16,+1-2 mar[20] | MUT |
|  | sAML | 47,XX,del(5)(q13q33),+8,+11,der(16)t(16;17)(q11.2;q21),-17[17]/46,idem,-4, t(6;10)(p21;p13),t(8;8)(q?11.2;q?),+mar[2]/46,XX[1] | MUT |
|  | sAML | 45,XX,del(3)(p14),-5,-7, add(17)(p11.2),+22[4]/46,XX[cp17] | WT |
|  | sAML | 45-46,XY,der(3;16)(q10;q10)[19],　del(5)(q22q35)[19],add(12)(p11.2)[19],add(12)(q13)[19],del(14)(q24q32)[19],-17[9],add(17)(q12)[9],-19[3],+add(21)(p12)[9],+22[10],+mar1[3][cp19]/46,XY[1] | WT |
|  | sAML | 46,XY,del(5)(q15q31)[11]/46,XY[9]. | WT |
|  | sAML | 46,XY,del(5)(q12q33),del(13)(q12q14)[18]/46,XY[2]. | WT |
|  | sAML | 34-44,XY,-1[20],del(2)(q21q31)[2],add(3)(q11.2)[20],-4[20],-5[19],add(5)(p15)[4],-6[6],-7[20],-8[20],+add(9)(p12)[20],-12[11],del(12)(q15)[9],-15[20]-16[4],-17[20]-18[5],add(19)(p13.3)[3],add(20)(q11.2)[6],+mar1[19],+mar2[19],+mar3[12],+mar4[20],+mar5[6] | WT |
|  | sAML | 44,XY,-3,del(5)(q22q35),-7,der(13;22)(q10;q10),+der(13;22)(q10;q10)[20] | WT |
|  | sAML | 44,XY,del(5)(q15q33),der(7;21)(q10;q10),der(12;15)(q10;q10)[20] | WT |
|  | sAML | 44-45,XX,del(5)(q13q33),-6,-10,-13,add(14)(q32),add(17)(p11.2),add(20)(p11.2),+r,+mar[cp3]/46,XX[6] | WT |
|  | sAML | 44-45,XY,add(1)(q44)[6],-5[15],del(5)(q13)[2],del(7)(q11.2q34)[14],t(8;18)(q13;q21)[15],add(13)(p13)[16],add(16)(q24)[15],add(17)(p11.2)[17],add(21)(q22)[15],+1-2mar[6][cp18]/46,XY[2] | WT |
|  | sAML | 44-47,XX,del (5)(q13q33), add(7)(q22), i(8)(q10),-9,add(11)(q23),-12,der(15)t(12;?;15)(q11;?;p11.2),-16,add(17)(p11.2),+mar1, +mar2[cp15]/46,XX[5] | WT |
|  | sAML | 46,XX,del(5)/45,idem,-16[18] | WT |
|  | sAML | 90-92,XXYY,del(5)(q13q33),-7,+13,+13,der(15)t(9;15)-21,-21,+mar1,+mar | WT |
