# Supplementary material for: Recurrent genetic defects on chromosome 5q in myeloid neoplasms
Source: Oncotarget. 2016 Dec 23;8(4):6483–95. doi: 10.18632/oncotarget.14130 (PMC5351647; doi:10.18632/oncotarget.14130)
Supplement: Supplementary file 3 [file oncotarget-08-6483-s003.docx]

**Supplementary Table 3.　 Mutations on chr5**

| Gene | Mutation | RefSeq | AA change | Position |
| --- | --- | --- | --- | --- |
| *PARP8* | Missense | NM_024615 | p.F578L | 5q11.2 |
| *ARL15* | Missense | NM_019087 | p.H129Y | 5q11.2 |
| *SKIV2L2* | Missense | NM_015360 | p.P720T | 5q11.2 |
| *PPAP2A* | Missense | NM_176895 | p.Y222D | 5q11.2 |
| *DDX4* | Nonsense | NM_001166534 | p.W521X | 5q11.2 |
| *MIER3* | Missense | NM_152622 | p.R287G | 5q11.2 |
| *ACTBL2* | Missense | NM_001017992 | p.S266P | 5q11.2 |
| *ZSWIM6* | Missense | ENST00000252744 | p.R105C | 5q12.1 |
| *HTR1A* | Missense | NM_000524 | p.N300T | 5q12.3 |
| *SREK1* | Missense | NM_001077199 | p.Q330H | 5q12.3 |
| *MAST4* | Missense | NM_001164664 | p.K1799R | 5q12.3 |
| *MAP1B* | Missense | NM_005909 | p.F926L | 5q13.2 |
| *MAP1B* | Missense | NM_005909 | p.T2166I | 5q13.2 |
| *MRPS27* | Nonsense | NM_015084 | p.Q396X | 5q13.2 |
| *TMEM174* | Missense | NM_153217 | p.T203M | 5q13.2 |
| *RGNEF* | Missense | NM_001080479 | p.Q469K | 5q13.2 |
| *ENC1* | Missense | NM_003633 | p.R471S | 5q13.3 |
| *ENC1* | Splice | NM_003633 | ¯ | 5q13.3 |
| *HEXB* | Nonsense | NM_000521 | p.W236X | 5q13.3 |
| *GFM2* | Frameshift | NM_032380 | p.FE609 In_Frame_Del | 5q13.3 |
| *LOC100130128* | Missense | XM_001715663 | p.T148I | 5q13.3 |
| *POC5* | Missense | NM_001099271 | p.A429V | 5q13.3 |
| *F2RL1* | Missense | NM_005242 | p.T25I | 5q13.3 |
| *AGGF1* | Missense | NM_018046 | p.A201V | 5q13.3 |
| *CMYA5* | Missense | NM_153610 | p.E1022K | 5q14.1 |
| *CMYA5* | Missense | NM_153610 | p.H1796Y | 5q14.1 |
| *CMYA5* | Missense | NM_153610 | p.P3275S | 5q14.1 |
| *ANKRD34B* | Missense | NM_001004441 | p.H90Y | 5q14.1 |
| *VCAN* | Missense | NM_004385 | p.P858T | 5q14.3 |
| *GPR98* | Missense | NM_032119 | p.D601N | 5q14.3 |
| *GPR98* | Missense | NM_032119 | p.R2090H | 5q14.3 |
| *GPR98* | Missense | NM_032119 | p.R2090H | 5q14.3 |
| *GPR98* | Missense | NM_032119 | p.A4495V | 5q14.3 |
| *LIX1* | Missense | NM_153234 | p.I211N | 5q15 |
| *ST8SIA4* | Missense | NM_005668 | p.H122R | 5q21.1 |
| *SLCO4C1* | Missense | NM_180991 | p.S190L | 5q21.1 |
| *CAMK4* | Missense | NM_001744 | p.K199R | 5q22.1 |
| *APC* | Missense | NM_000038 | p.G126E | 5q22.2 |
| *APC* | Missense | NM_000038 | p.N389S | 5q22.2 |
| *APC* | Missense | NM_000038 | p.S2601R | 5q22.2 |
| *APC* | Missense | NM_000038 | p.R2714C | 5q22.2 |
| *LOC100131040* | Missense | XM_001715034 | p.S25G | 5q23.1 |
| *LOC100130087* | Missense | XM_001715088 | p.G116R | 5q23.1 |
| *AP3S1* | Frameshift | NM_001284 | p.K41fs | 5q23.1 |
| *AP3S1* | Frameshift | NM_001284 | p.K41fs | 5q23.1 |
| *AP3S1* | Frameshift | NM_001284 | p.K41fs | 5q23.1 |
| *SEMA6A* | Missense | CCDS47256.1 | p.Y147C | 5q23.1 |
| *FAM170A* | Missense | NM_001163991 | p.S15F | 5q23.1 |
| *FAM170A* | Nonsense | NM_182761 | p.R92X | 5q23.1 |
| *SNCAIP* | Missense | NM_005460 | p.E756K | 5q23.2 |
| *PPIC* | Missense | NM_000943 | p.N142S | 5q23.2 |
| *ALDH7A1* | Missense | NM_001182 | p.G115V | 5q23.2 |
| *MEGF10* | Missense | NM_032446 | p.W66L | 5q23.2 |
| *SLC27A6* | Missense | NM_001017372 | p.R333H | 5q23.3 |
| *P4HA2* | Missense | NM_004199 | p.I150N | 5q31.1 |
| *IRF1* | Nonsense | CCDS4155.1 | p.Y65X | 5q31.1 |
| *AFF4* | Missense | NM_014423 | p.K781Q | 5q31.1 |
| *HSPA4* | Nonsense | NM_002154 | p.L586X | 5q31.1 |
| *VDAC1* | Missense | NM_003374 | p.L275F | 5q31.1 |
| *TCF7* | Missense | NM_003202 | p.S356F | 5q31.1 |
| *CDKL3* | Missense | ENST00000265334 | p.E490K | 5q31.1 |
| *LOC153328* | Nonsense | NM_145282 | p.E7X | 5q31.1 |
| *TGFBI* | Missense | NM_000358 | p.P612S | 5q31.1 |
| *TRPC7* | Splice | ENST00000265193 | ¯ | 5q31.1 |
| *NPY6R* | Missense | ENST00000290426 | p.K63M | 5q31.2 |
| *KDM3B* | Missense | NM_016604 | p.H83N | 5q31.2 |
| *KDM3B* | Frameshift | NM_016604 | p.Q827fs | 5q31.2 |
| *KDM3B* | Missense | NM_016604 | p.R938H | 5q31.2 |
| *ETF1* | Missense | NM_004730 | p.F131V | 5q31.2 |
| *LRRTM2* | Missense | NM_015564 | p.R170G | 5q31.2 |
| *LRRTM2* | Missense | NM_015564 | p.Q144E | 5q31.2 |
| *MATR3* | Missense | NM_001194955 | p.R595T | 5q31.2 |
| *PSD2* | Missense | NM_032289 | p.G363E | 5q31.2 |
| *PURA* | Missense | NM_005859 | p.Q285K | 5q31.2 |
| *SRA1* | Missense | NM_001035235 | p.A221V | 5q31.3 |
| *PCDHA1* | Missense | NM_018900 | p.A567T | 5q31.3 |
| *PCDHA4* | Missense | NM_018907 | p.S432L | 5q31.3 |
| *PCDHA7* | Missense | NM_018910 | p.A706V | 5q31.3 |
| *PCDHA8* | Missense | NM_018911 | p.P618T | 5q31.3 |
| *uc003lhy.1* | Frameshift | ENST00000319589 | p.G175fs | 5q31.3 |
| *uc003lhy.1* | Frameshift | ENST00000319589 | p.G175fs | 5q31.3 |
| *PCDHA12* | Missense | NM_018903 | p.L21R | 5q31.3 |
| *PCDHAC1* | Missense | NM_018898 | p.Q218K | 5q31.3 |
| *PCDHAC1* | Missense | NM_031882 | p.R622C | 5q31.3 |
| *PCDHB1* | Missense | NM_013340 | p.V714I | 5q31.3 |
| *PCDHB3* | Missense | NM_018937 | p.P221L | 5q31.3 |
| *PCDHB6* | Frameshift | NM_018939 | p.N86fs | 5q31.3 |
| *PCDHB6* | Missense | NM_018939 | p.D158E | 5q31.3 |
| *PCDHB7* | Missense | NM_018940 | p.R229C | 5q31.3 |
| *PCDHB8* | Missense | NM_019120 | p.L714P | 5q31.3 |
| *PCDHB16* | Missense | NM_020957 | p.T405M | 5q31.3 |
| *PCDHB9* | Missense | NM_019119 | p.T483M | 5q31.3 |
| *PCDHB9* | Missense | NM_019119 | p.T749I | 5q31.3 |
| *PCDHB10* | Missense | NM_018930 | p.G532S | 5q31.3 |
| *PCDHB11* | Missense | NM_018931 | p.S375P | 5q31.3 |
| *PCDHB18* | Missense | ENST00000274705 | p.Y435H | 5q31.3 |
| *PCDHB15* | Missense | NM_018935 | p.V439M | 5q31.3 |
| *PCDHB15* | Missense | NM_018935 | p.V635M | 5q31.3 |
| *PCDHGA1* | Missense | CCDS34256.1 | p.T690I | 5q31.3 |
| *PCDHGA3* | Missense | NM_018916 | p.R202H | 5q31.3 |
| *PCDHGA5* | Missense | NM_018918 | p.G374V | 5q31.3 |
| *PCDHGA6* | Missense | NM_018919 | p.G204V | 5q31.3 |
| *PCDHGA9* | Missense | NM_018921 | p.A28V | 5q31.3 |
| *PCDHGA10* | Frameshift | NM_018913 | p.E342 In_Frame_Del | 5q31.3 |
| *PCDHGA12* | Missense | NM_003735 | p.G217V | 5q31.3 |
| *PCDHGC3* | Frameshift | NM_032402 | p.T659fs | 5q31.3 |
| *PCDHGC4* | Missense | NM_018928.2 | p.P452A | 5q31.3 |
| *HDAC3* | Missense | NM_003883 | p.N148S | 5q31.3 |
| *RELL2* | Missense | NM_173828 | p.T110N | 5q31.3 |
| *PCDH1* | Missense | NM_032420 | p.F612S | 5q31.3 |
| *KIAA0141* | Missense | NM_014773 | p.I498T | 5q31.3 |
| *PCDHGA1* | Missense | NM_031993 | p.A656T | 5q31.3 |
| *SH3RF2* | Missense | NM_152550 | p.V179M | 5q32 |
| *LARS* | Missense | NM_020117 | p.A177G | 5q32 |
| *PPP2R2B* | Missense | NM_181676 | p.R374C | 5q32 |
| *STK32A* | Nonsense | ENST00000306304 | p.R268X | 5q32 |
| *DPYSL3* | Missense | NM_001387 | p.I141V | 5q32 |
| *SPINK5* | Missense | NM_001127699 | p.R853Q | 5q32 |
| *SH3TC2* | Missense | NM_024577 | p.A1090V | 5q32 |
| *SH3TC2* | Missense | NM_024577 | p.R1012Q | 5q32 |
| *CSNK1A1* | Missense | NM_001025105 | p.E98K | 5q32 |
| *ENSG00000214485* | Missense | ENST00000398410 | p.K189T | 5q32 |
| *PDGFRB* | Missense | NM_002609 | p.R604C | 5q32 |
| *CD74* | Missense | NM_001025159 | p.P206S | 5q32 |
| *CD74* | Missense | NM_001025159 | p.R10W | 5q32 |
| *SYNPO* | Missense | NM_007286 | p.I245L | 5q33.1 |
| *RBM22* | Missense | NM_018047 | p.P32Q | 5q33.1 |
| *ANXA6* | Missense | NM_001155 | p.G266V | 5q33.1 |
| *FAT2* | Missense | NM_001447 | p.L3279V | 5q33.1 |
| *FAT2* | Frameshift | NM_001447 | p.P2583fs | 5q33.1 |
| *FAT2* | Missense | NM_001447 | p.E1561K | 5q33.1 |
| *G3BP1* | Missense | NM_005754 | p.G12R | 5q33.1 |
| *G3BP1* | Missense | NM_198395 | p.R370P | 5q33.1 |
| *GLRA1* | Missense | NM_000171 | p.T6I | 5q33.1 |
| *NMUR2* | Missense | NM_020167 | p.A154V | 5q33.1 |
| *NMUR2* | Missense | NM_020167 | p.V140I | 5q33.1 |
| *NMUR2* | Missense | NM_020167 | p.R41C | 5q33.1 |
| *LARP1* | Missense | NM_033551 | p.P487S | 5q33.2 |
| *ADAM19* | Missense | NM_023038 | p.H599N | 5q33.3 |
| *ADAM19* | Nonsense | NM_033274 | p.E75X | 5q33.3 |
| *PWWP2A* | Missense | NM_052927 | p.A516T | 5q33.3 |
| *ATP10B* | Missense | NM_025153 | p.Y1181H | 5q34 |
| *ATP10B* | Missense | NM_025153 | p.K139R | 5q34 |
| *GABRG2* | Missense | NM_198904 | p.V332F | 5q34 |
| *MAT2B* | Nonsense | NM_013283 | p.W250X | 5q34 |
| *ODZ2* | Missense | ENST00000388903 | p.G2541V | 5q34 |
| *RARS* | Missense | NM_002887 | p.N181S | 5q34 |
| *SLIT3* | Missense | NM_003062 | G1440S | 5q34 |
| *DOCK2* | Splice | NM_004946 | ¯ | 5q35.1 |
| *DOCK2* | Missense | NM_004946 | p.I417V | 5q35.1 |
| *NPM1* | Frameshift | NM_002520 | p.L287fs | 5q35.1 |
| *NPM1* | Frameshift | NM_002520 | p.L287fs | 5q35.1 |
| *NPM1* | Frameshift | NM_002520 | p.W288fs | 5q35.1 |
| *NPM1* | Frameshift | NM_002520 | p.W288fs | 5q35.1 |
| *NPM1* | Frameshift | NM_002520 | p.W288fs | 5q35.1 |
| *NPM1* | Frameshift | NM_002520 | p.W288fs | 5q35.1 |
| *NPM1* | Frameshift | NM_002520 | p.W288fs | 5q35.1 |
| *NPM1* | Frameshift | NM_002520 | p.W288fs | 5q35.1 |
| *NPM1* | Frameshift | NM_002520 | p.W288fs | 5q35.1 |
| *NPM1* | Frameshift | NM_002520 | p.W288fs | 5q35.1 |
| *NPM1* | Frameshift | NM_002520 | p.W288fs | 5q35.1 |
| *NPM1* | Frameshift | NM_002520 | p.W288fs | 5q35.1 |
| *NPM1* | Frameshift | NM_002520 | p.W288fs | 5q35.1 |
| *NPM1* | Frameshift | NM_002520 | p.W288fs | 5q35.1 |
| *NPM1* | Frameshift | NM_002520 | p.W288fs | 5q35.1 |
| *NPM1* | Frameshift | NM_002520 | p.W288fs | 5q35.1 |
| *NPM1* | Frameshift | NM_002520 | p.W288fs | 5q35.1 |
| *NPM1* | Frameshift | NM_002520 | p.W288fs | 5q35.1 |
| *NPM1* | Frameshift | NM_002520 | p.W288fs | 5q35.1 |
| *NPM1* | Frameshift | NM_002520 | p.W288fs | 5q35.1 |
| *NPM1* | Frameshift | NM_002520 | p.W288fs | 5q35.1 |
| *NPM1* | Frameshift | NM_002520 | p.W288fs | 5q35.1 |
| *NPM1* | Frameshift | NM_002520 | p.W288fs | 5q35.1 |
| *NPM1* | Frameshift | NM_002520 | p.W288fs | 5q35.1 |
| *NPM1* | Frameshift | NM_002520 | p.W288fs | 5q35.1 |
| *NPM1* | Frameshift | NM_002520 | p.W288fs | 5q35.1 |
| *NPM1* | Frameshift | NM_002520 | p.W288fs | 5q35.1 |
| *NPM1* | Frameshift | NM_002520 | p.W288fs | 5q35.1 |
| *NPM1* | Frameshift | NM_002520 | p.W288fs | 5q35.1 |
| *NPM1* | Frameshift | NM_002520 | p.W288fs | 5q35.1 |
| *NPM1* | Frameshift | NM_002520 | p.W288fs | 5q35.1 |
| *NPM1* | Frameshift | NM_002520 | p.W288fs | 5q35.1 |
| *NPM1* | Frameshift | NM_002520 | p.W288fs | 5q35.1 |
| *NPM1* | Frameshift | NM_002520 | p.W288fs | 5q35.1 |
| *NPM1* | Frameshift | NM_002520 | p.W288fs | 5q35.1 |
| *NPM1* | Frameshift | NM_002520 | p.W288fs | 5q35.1 |
| *NPM1* | Frameshift | NM_002520 | p.W288fs | 5q35.1 |
| *NPM1* | Frameshift | NM_002520 | p.W288fs | 5q35.1 |
| *NPM1* | Frameshift | NM_002520 | p.W288fs | 5q35.1 |
| *NPM1* | Frameshift | NM_002520 | p.W288fs | 5q35.1 |
| *NPM1* | Frameshift | NM_002520 | p.W288fs | 5q35.1 |
| *NPM1* | Frameshift | NM_002520 | p.W288fs | 5q35.1 |
| *NPM1* | Frameshift | NM_002520 | p.W288fs | 5q35.1 |
| *NPM1* | Frameshift | NM_002520 | p.W288fs | 5q35.1 |
| *NPM1* | Frameshift | NM_002520 | p.W288fs | 5q35.1 |
| *NPM1* | Frameshift | NM_002520 | p.W288fs | 5q35.1 |
| *NPM1* | Frameshift | NM_002520 | p.W288fs | 5q35.1 |
| *NPM1* | Frameshift | NM_002520 | p.W288fs | 5q35.1 |
| *NPM1* | Frameshift | NM_002520 | p.W288fs | 5q35.1 |
| *NPM1* | Frameshift | NM_002520 | p.W288fs | 5q35.1 |
| *STK10* | Missense | NM_005990 | p.K65E | 5q35.1 |
| *STK10* | Missense | NM_005990 | p.E57A | 5q35.1 |
| *ENSG00000204762* | Missense | ENST00000377334 | p.G9S | 5q35.1 |
| *DUSP1* | Missense | NM_004417 | p.N351S | 5q35.1 |
| *DRD1* | Missense | NM_000794 | p.Y131H | 5q35.2 |
| *SFXN1* | Frameshift | NM_022754 | p.D209fs | 5q35.2 |
| *C5orf25* | Missense | NM_198567 | p.T33I | 5q35.2 |
| *C5orf25* | Missense | NM_198567 | p.T33I | 5q35.2 |
| *C5orf25* | Missense | NM_198567 | p.R195C | 5q35.2 |
| *C5orf25* | Frameshift | NM_198567 | p.L426fs | 5q35.2 |
| *CLTB* | Nonsense | NM_001834 | p.Q146X | 5q35.2 |
| *CDHR2* | Missense | NM_017675.3 | p.P1004L | 5q35.2 |
| *GPRIN1* | Missense | NM_052899 | p.C428W | 5q35.2 |
| *UNC5A* | Missense | NM_133369 | p.R773W | 5q35.2 |
| *UIMC1* | Missense | NM_001199297 | p.K616R | 5q35.2 |
| *NSD1* | Missense | NM_172349 | p.R71K | 5q35.3 |
| *RAB24* | Missense | NM_001031677 | p.A176D | 5q35.3 |
| *LMAN2* | Missense | NM_006816 | p.Q65H | 5q35.3 |
| *PDLIM7* | Nonsense | NM_005451 | p.W360X | 5q35.3 |
| *DDX41* | Missense | NM_016222 | p.R525H | 5q35.3 |
| *DDX41* | Missense | NM_016222 | p.R525H | 5q35.3 |
| *DDX41* | Missense | NM_016222 | p.R525H | 5q35.3 |
| *DDX41* | Missense | NM_016222 | p.R525H | 5q35.3 |
| *DDX41* | Splice | NM_016222.2 | ¯ | 5q35.3 |
| *LOC100128340* | Missense | XM_001721455 | p.P35S | 5q35.3 |
| *GRM6* | Missense | NM_000843 | p.G630D | 5q35.3 |
| *GRM6* | Missense | NM_000843 | p.G508D | 5q35.3 |
| *RUFY1* | Missense | NM_025158 | p.L579V | 5q35.3 |
| *HNRNPH1* | Missense | NM_005520 | p.A385V | 5q35.3 |
| *HNRNPH1* | Missense | NM_005520 | p.P19S | 5q35.3 |
| *ENSG00000204661* | Missense | ENST00000376976 | p.S41N | 5q35.3 |
| *MGAT4B* | Missense | NM_054013 | p.P187L | 5q35.3 |
| *TBC1D9B* | Nonsense | NM_015043 | p.R555X | 5q35.3 |
| *RASGEF1C* | Missense | ENST00000274820 | p.C231Y | 5q35.3 |
| *GFPT2* | Missense | NM_005110 | p.R613P | 5q35.3 |
| *CNOT6* | Missense | NM_015455 | p.R335L | 5q35.3 |
| *FLT4* | Missense | NM_182925 | p.R589H | 5q35.3 |
| *OR2Y1* | Nonsense | NM_001001657 | p.W20 | 5q35.3 |
| *TRIM7* | Missense | NM_203293 | p.R450W | 5q35.3 |
